# Ubiquitination of the protocadherin-γA3 variable cytoplasmic domain modulates cell-cell interaction

**DOI:** 10.3389/fcell.2023.1261048

**Published:** 2023-09-18

**Authors:** Albert Ptashnik, Nicole LaMassa, Aliya Mambetalieva, Emily Schnall, Mike Bucaro, Greg R. Phillips

**Affiliations:** ^1^ Department of Biology, College of Staten Island, City University of New York, New York, NY, United States; ^2^ PhD Program in Biology, Subprogram in Neuroscience, CUNY Graduate Center, New York, NY, United States; ^3^ Center for Developmental Neuroscience, College of Staten Island, City University of New York, New York, NY, United States

**Keywords:** endosome, cell adhesion, self-avoidance, endocytosis, pseudophosphorylation

## Abstract

The family of ∼60 clustered protocadherins (Pcdhs) are cell adhesion molecules encoded by a genomic locus that regulates expression of distinct combinations of isoforms in individual neurons resulting in what is thought to be a neural surface “barcode” which mediates same-cell interactions of dendrites, as well as interactions with other cells in the environment. Pcdh mediated same-cell dendrite interactions were shown to result in avoidance while interactions between different cells through Pcdhs, such as between neurons and astrocytes, appear to be stable. The cell biological mechanism of the consequences of Pcdh based adhesion is not well understood although various signaling pathways have been recently uncovered. A still unidentified cytoplasmic regulatory mechanism might contribute to a “switch” between avoidance and adhesion. We have proposed that endocytosis and intracellular trafficking could be part of such a switch. Here we use “stub” constructs consisting of the proximal cytoplasmic domain (lacking the constant carboxy-terminal domain spliced to all Pcdh-γs) of one Pcdh, Pcdh-γA3, to study trafficking. We found that the stub construct traffics primarily to Rab7 positive endosomes very similarly to the full length molecule and deletion of a substantial portion of the carboxy-terminus of the stub eliminates this trafficking. The intact stub was found to be ubiquitinated while the deletion was not and this ubiquitination was found to be at non-lysine sites. Further deletion mapping of the residues required for ubiquitination identified potential serine phosphorylation sites, conserved among Pcdh-γAs, that can reduce ubiquitination when pseudophosphorylated and increase surface expression. These results suggest Pcdh-γA ubiquitination can influence surface expression which may modulate adhesive activity during neural development.

## Introduction

Development and extension of axons and dendrites in their proper trajectories involves the interaction of these processes with surrounding cells via the action of cell adhesion molecules. There are many families of cell adhesion molecules that participate in neural development. Of recent interest is a subfamily of cadherins called the protocadherins, members of which appear to mediate specific events in the development of neural circuits. The protocadherins are further divided into families of clustered and non-clustered protocadherins. For the clustered protocadherins (referred here as Pcdhs) the genomic arrangement of ∼60 different isoforms (Wu and Maniatis, 1999), each with homophilic specificity, allows distinct combinations of these isoforms to be expressed in individual neurons, generating what has been termed an adhesive “barcode” for each neuron (Thu et al., 2014; Rubinstein et al., 2015; Rubinstein et al., 2017; Goodman et al., 2016a; Goodman et al., 2016b; Goodman et al., 2017; Brasch et al., 2019; Canzio and Maniatis, 2019). There are 3 different genomic Pcdh subclusters (α, β, γ) in which “variable” exons that encode an extracellular, transmembrane and proximal cytoplasmic domain can be spliced to downstream exons that encode a common distal cytoplasmic domain (Wu and Maniatis, 1999).

How this barcode functions is not completely understood at the level of neural circuits as well as at the cellular level. It has been shown that the barcode can participate in establishment of synaptic connectivity (Weiner et al., 2005; Garrett and Weiner, 2009; Molumby et al., 2017; Steffen et al., 2021; Meltzer et al., 2023). The enormous number of permutations (Schreiner and Weiner, 2010; Thu et al., 2014) possible due to stochastic epigenetic regulation (Tasic et al., 2002; Kawaguchi et al., 2008; Toyoda et al., 2014) of Pcdh expression in each neuron is consistent with a role in synaptic connectivity. Another function for the barcode was shown to be in self-avoidance in certain cell types that exhibit planar dendritic trees as interference with the Pcdh-γ gene cluster causes abnormal clumping of same-cell dendrites but no effect on interaction of dendrites from adjacent cells (Lefebvre et al., 2012; Ing-Esteves et al., 2018). Axons are also affected in Pcdh mutants as loss of Pcdhs caused abnormal olfactory glomeruli development (Hasegawa et al., 2012; Mountoufaris et al., 2017) as well as defective retinogeniculate (Meguro et al., 2015) or serotonergic (Katori et al., 2009; Katori et al., 2017) projections. These results reflect a repulsive interaction mediated by Pcdhs. Still another function for Pcdhs was shown to be interaction of dendrites with astrocytes in which an adhesive interaction was implicated (Garrett and Weiner, 2009; Molumby et al., 2016).

Pcdhs have adhesive activity in their extracellular domains, but were initially found to exhibit weak adhesion in the standard assay for cell aggregation in mouse L-cells (Obata et al., 1995). Later they were found to exhibit robust cell aggregation in K562 cells (Schreiner and Weiner, 2010), particularly when the cytoplasmic domains were deleted, which was found to increase cell surface expression (Fernández-Monreal et al., 2009). One fundamental question that arises is: what is the cellular mechanism by which cell adhesion molecules, which typically bind cells together, can mediate the opposite function observed in dendritic self-avoidance, the separation of membranes? Such differences in activity may involve regulation of the Pcdh cytoplasmic domain which was shown to modulate surface delivery and promote trafficking in endosomes (O’Leary et al., 2011). Based on our studies of Pcdh-γA3, which is a member of the Pcdh-γA subfamily of Pcdhs, the largest Pcdh subfamily with 12 isoforms in mouse, we have hypothesized that regulation of endocytosis may be a mechanism for downregulation of adhesion mediated by Pcdhs (Phillips et al., 2017), as has been observed for other cell adhesion molecules [reviewed in (Niño et al., 2019)]. We have shown that intracellular trafficking is largely mediated by the variable portion of the cytoplasmic domain (VCD) for Pcdh-γA3 (O’Leary et al., 2011).

Here we use “stub” constructs derived from Pcdh-γA3, that lack the extracellular domain and the constant cytoplasmic domain, and show they exhibit trafficking primarily to Rab7 positive endosomes similar to the full length molecule. The stub was found to be ubiquitinated at a non-lysine site in the cytoplasmic domain that was dependent on a segment in the cytoplasmic domain predicted to contain 2 serine phosphorylation sites conserved within 11 out of 12 genes comprising the Pcdh-γA family in mouse. Pseudophosphorylation of both these sites reduced ubiquitination and promoted surface delivery of the stub. These results suggest modulation of Pcdh surface delivery by ubiquitination of the cytoplasmic domain could alter adhesive activity during development.

## Results

### Pcdh trafficking to Rab7 positive endosomes

**FIGURE 1:**
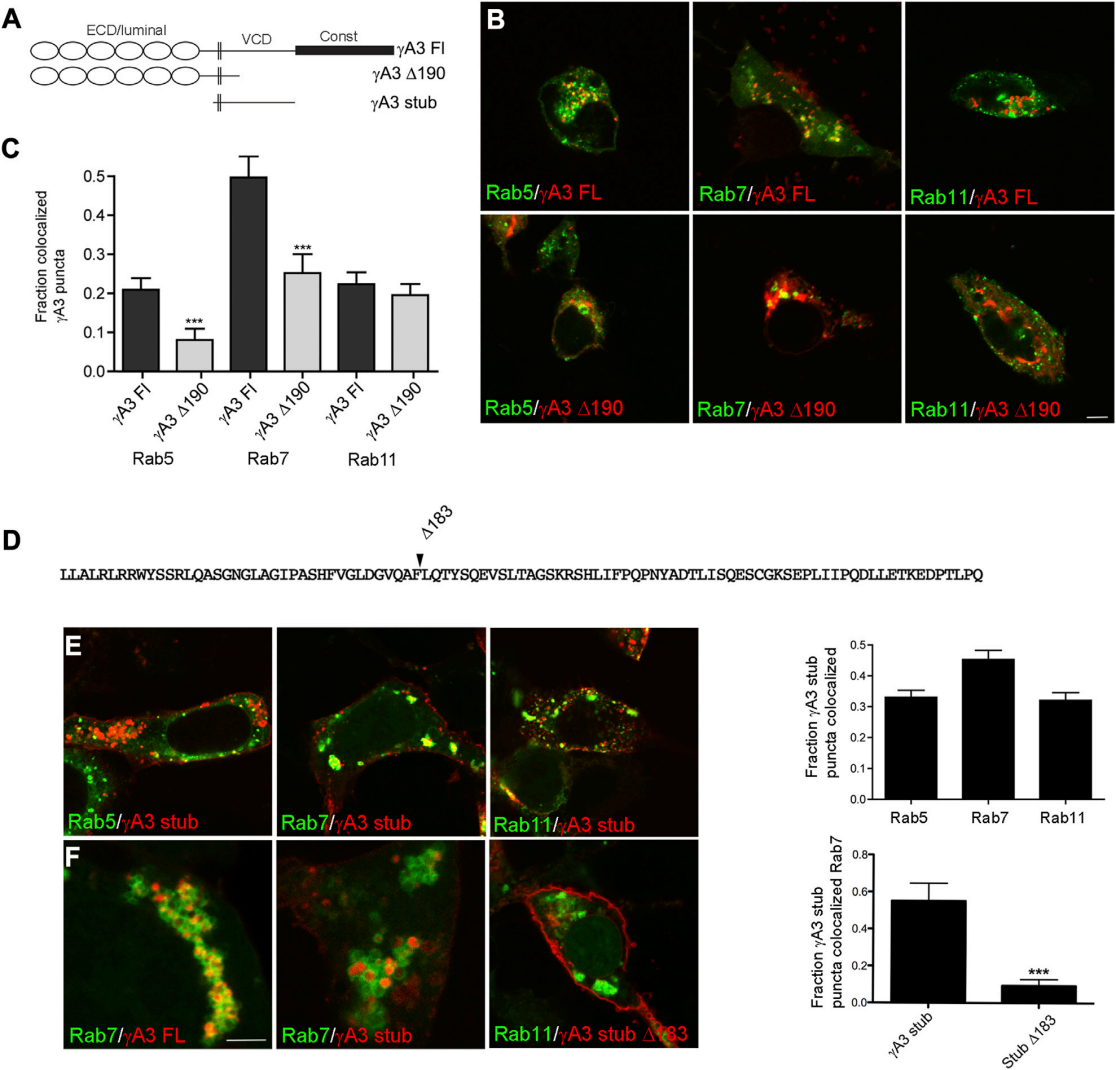
The Pcdh-γA3 VCD is sufficient to promote trafficking to the late endosome. **(A)** Diagram of Pcdh-γA3 constructs. **(B)** Pcdh-γA3 full length (γA3 FL) or the cytoplasmic deletion (γA3 Δ190), fused to RFP, were cotransfected with different Rab-GFP markers. **(C)** Quantification of colocalization. γA3 FL preferentially colocalized with Rab7, a marker for the late endosome and this colocalization was significantly diminished for γA3 Δ190. Colocalization with Rab5, a marker for late endosomes, was also significantly diminished upon cytoplasmic deletion. Colocalization with Rab11, a marker for recycling endosomes, was unaffected by cytoplasmic deletion. **(D)** Sequence of the Pcdh-γA3 VCD which comprises the intracellular segment of the γA3 stub construct. Location of the Δ183 deletion in the γA3 stub is indicated. **(E)** The γA3 stub preferentially colocalizes with Rab7, similar to the full length molecule. **(F)** Full length Pcdh-γA3 and the γA3 stub are trafficked to the interior of Rab7 positive vesicles (left and middle panels). In contrast, γA3 stub Δ183 does not colocalize significantly with Rab7 positive vesicles. Bar = 5 μm in **(B,E)** and **(F)** right panel, 300 nm in **(F)** left and middle panels. ***p <0.001.

Previous results showed that Pcdh-γA3 traffics to intracellular compartments that resemble the late endosome at the electron microscopy level in HEK 293 cells (Fernández-Monreal et al., 2009; Hanson et al., 2010; O’Leary et al., 2011). To confirm the trafficking pathway for Pcdh-γA3, cells were co-transfected with a plasmid coding for the full length molecule or the cytoplasmic deletion, fused to RFP (γA3 FL and γA3 Δ190, respectively), together with plasmids coding for markers of early, late, recycling endosomes (Rab5, Rab7, or Rab11, respectively), fused to GFP (Figures 1A, B). Quantitative image analysis showed that γA3 FL was preferentially trafficked to Rab7 positive late endosomes. Rab5 (early endosomes) and Rab11 (recycling endosomes) exhibited less colocalization with γA3 FL (Figure 1C).

It was previously shown that deletion of the cytoplasmic domain including the constant domain and most of the VCD (γA3 Δ190) increases surface expression with a concomitant decrease in intracellular trafficking (Fernández-Monreal et al., 2009; Schreiner and Weiner, 2010). Consistently, γA3 Δ190 exhibited significantly decreased colocalization with Rab7 positive late endosomes as well as Rab5 positive early endosomes (Figure 1C). Thus Pcdh-γA3 is enriched mostly in late endosomes and that this localization requires the cytoplasmic domain.

Because previous studies showed that Pcdh-γA3 intracellular trafficking is mediated primarily by the VCD portion of the cytoplasmic domain, we used stub constructs of this Pcdh (γA3 stub, Figures 1A, D) that lack the extracellular and constant cytoplasmic domains to study determinants of trafficking in more detail. A stub construct in which most of the cytoplasmic domain was deleted was also prepared (γA3 stub Δ183, Figure 1D). Cells cotransfected with the γA3 stub and the different Rab markers exhibited similar results as the full length constructs in that the γA3 stub colocalized most strongly with Rab7 and to a lesser extent with Rab5 and Rab11 (Figure 1E). Higher magnification images revealed that both γA3 FL and the γA3 stub localized within the interior of Rab 7 positive vesicles (Figure 1F). In contrast, γA3 stub Δ183 no longer targeted to Rab 7 positive vesicles and was more readily found on the cell surface (Figure 1F). Thus the γA3 stub contains most of the endosomal trafficking determinants for Pcdh-γA3 although it is possible that the constant domain could also influence trafficking to a certain extent.

### Ubiquitination is associated with endosomal trafficking

**FIGURE 2:**
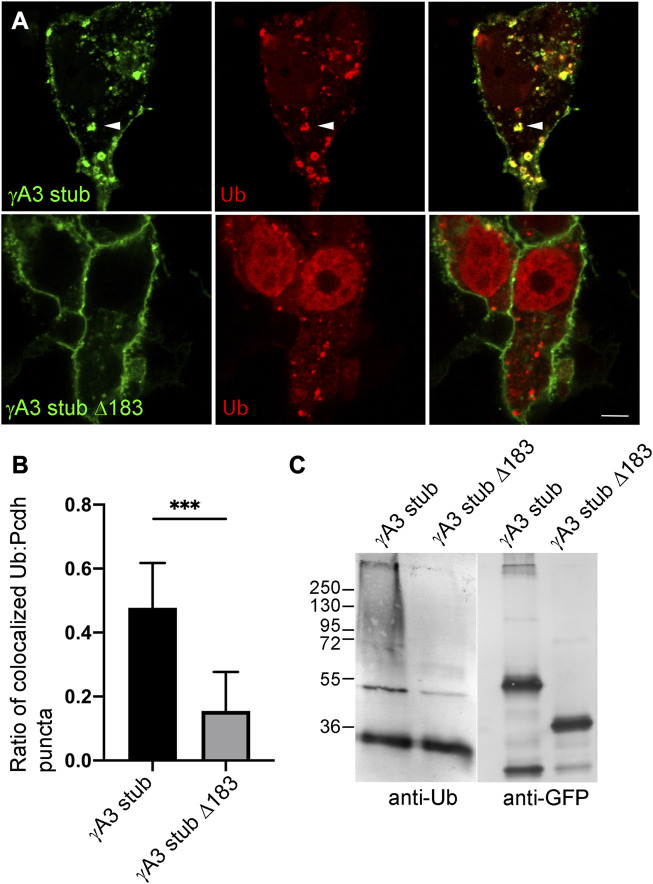
Ubiquitination of the γA3 stub is associated with intracellular trafficking. **(A)** γA3-GFP stub puncta colocalize with ubiquitin-RFP (top, arrowheads) while γA3 stub Δ183 (bottom) exhibits less colocalization with ubiquitin-RFP as quantified in **(B) (C)** γA3 stub GFP immunoprecipitates probed with anti-ubiquitin exhibit a high molecular weight smear that was not present in immunoprecipitates of γA3 stub Δ183 (left). Probing with anti-GFP reveals the equivalent expression levels of γA3 stub and γA3 stub Δ183 (right). ***p <0.001.

Ubiquitination of receptors involved in neural development, including cell adhesion molecules, has been shown to be associated with endocytosis, endosomal trafficking and downregulation of activity. We asked whether the γA3 stub was modified by ubiquitin and whether this was associated with intracellular trafficking. Cells were transfected with γA3 stub or γA3 stub Δ183, fused to GFP, together with Ubiquitin-RFP (Figures 2A, B). While γA3 stub colocalized extensively with ubiquitin in cotransfected cells, γA3 stub Δ183 showed substantially less colocalization with ubiquitin (Figures 2A, B). In addition, lysates transfected with γA3 stub or γA3 stub Δ183 were immunoprecipitated with anti-GFP antibodies and probed with antibodies to ubiquitin. An anti-ubiquitin smear was observed in γA3 stub immunoprecipitates that was not found in stub Δ183 immunoprecipitates (Figure 2C). Thus a site within the VCD of Pcdh-γA3 controls surface expression and is modified by ubiquitin.

**FIGURE 3:**
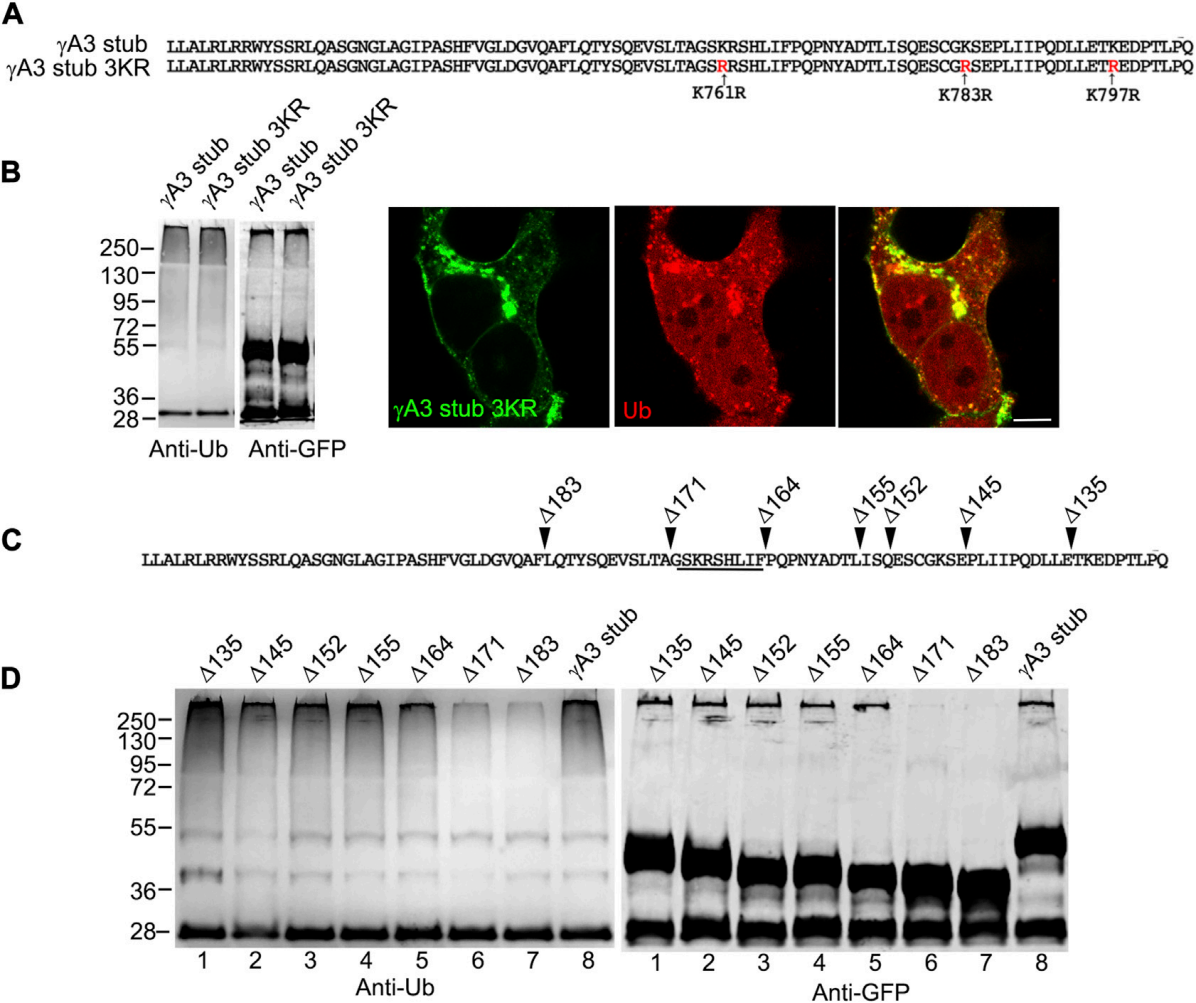
A segment of the Pcdh-γA3 VCD regulates ubiquitination. **(A)** Sequence of the γA3 VCD. The indicated lysines were mutated to arginine in γA3 stub 3KR. **(B)** Both γA3 stub and γA3 stub 3KR exhibit a similar ubiquitin smear when probed with anti-ubiquitin antibodies at similar expression levels (left panels). γA3 stub 3KR shows colocalization with ubiquitin (right panels). **(C)** Deletions of the γA3 stub are indicated. **(D)** GFP precipitates probed with anti-ubiquitin show similar ubiquitin smears for the γA3 stub Δ135, Δ145, Δ152, Δ155 and Δ164 deletions, but reduced smears for the Δ171 and Δ183 deletions indicating the presence of a critical segment in the VCD [underlined in **(C)**] that regulates ubiquitination.

The γA3 stub contains 3 cytoplasmic lysines at positions 761, 783 and 797 (numbered according to their position in full length mouse Pcdh-γA3) that are candidates for ubiquitination (Figure 3A). Each of these lysines were mutated to arginine (γA3 stub 3KR) and the level of ubiquitination was analyzed by immunoprecipitation followed by Western blot with anti-ubiquitin. Mutation of the lysines resulted in ubiquitination levels similar to that observed in the intact γA3 stub and similar colocalization with ubiquitin (Figure 3B).

### A 9 amino acid segment in the γA3 stub regulates ubiquitination and trafficking

**FIGURE 4:**
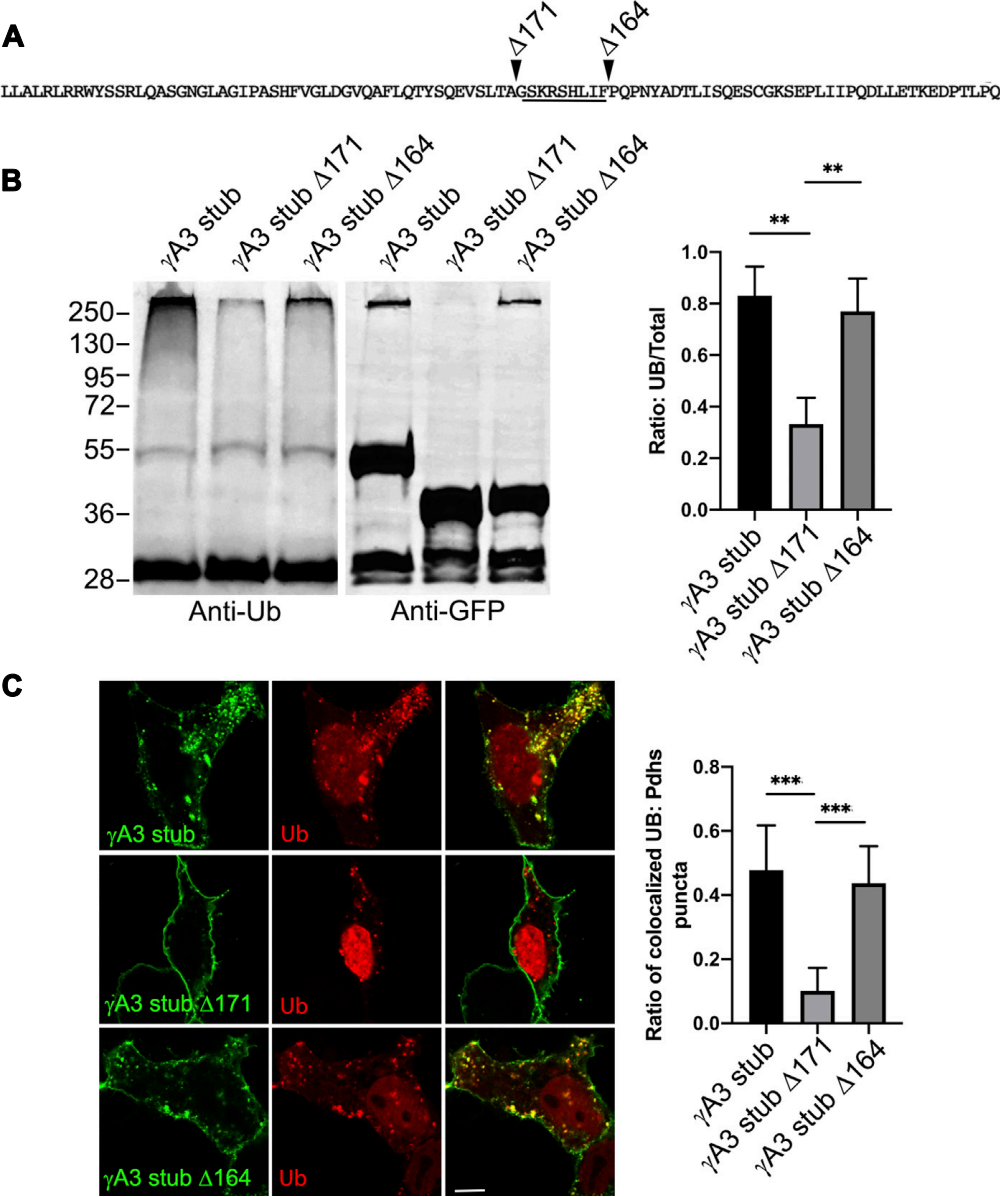
Quantification of γA3 stub, γA3 stub Δ164 and γA3 stub Δ171 ubiquitination. **(A)** Pcdh-γA3 VCD sequence with indicated deletions analyzed in the stub backbone. The segment required for ubiquitination is underlined. **(B)** γA3 stub, γA3 stub Δ164 and γA3 stub Δ171 immunoprecipitates were probed for anti-ubiquitin and GFP. Quantification of ubiquitin smear relative to GFP is shown at right. **p <0.005 **(C)** Cotransfection of γA3 stub, γA3 stub Δ164 and γA3 stub Δ171 with ubiquitin-RFP. Quantification of colocalization is shown at right. ***p <0.001.

We sought to further map the determinants of trafficking and ubiquitination of the γA3 stub by generating series of deletion mutants (numbered according to the amino acids deleted from the carboxy terminus of full length mouse Pcdh-γA3, Figure 3C). Deletion of residues up to a 9 amino acid segment in the VCD (γA3 stub Δ164), preserved ubiquitination (Figure 3D, lane 5) that was reduced when the additional 9 residues were deleted (γA3 stub Δ171, Figure 3D, lane 6). This was examined by quantitative Western blot whereby the levels of ubiquitination of the γA3 stub and γA3 stub Δ164 were similar while γA3 stub Δ171 (Figure 4A) showed a >50% reduction in ubiquitination as compared to γA3 stub (Figure 4B). γA3 Stub Δ171 also lacked significant colocalization with ubiquitin as compared to γA3 stub and γA3 stub Δ164 (Figure 4C).

**FIGURE 5:**
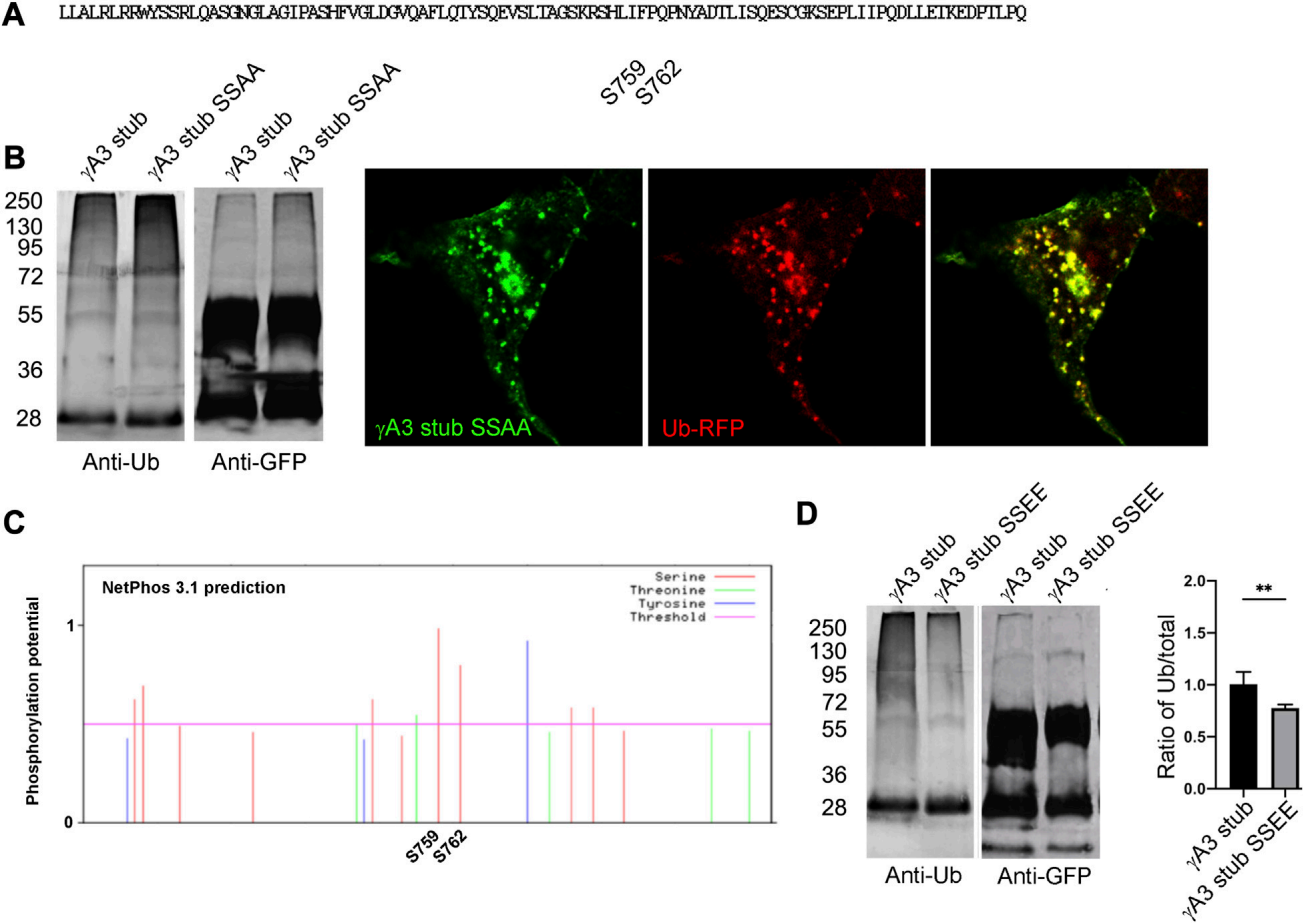
Serines 759 and 762 are not substrates for ubiquitination but may be phosphorylation sites that affect ubiquitination. **(A)** The indicated serines were mutated to alanine to generate γA3 stub SSAA. **(B)** γA3 stub and γA3 stub SSAA immunoprecipitates were probed for anti-ubiquitin and cotransfected with ubiquitin-RFP. Both exhibited similar ubiquitin smears and colocalization with ubiquitin. **(C)** Analysis of the VCD region of Pcdh-γA3 by NetPhos 3.1. Both serine 759 and 762 were the most likely within the VCD to be substrates for phosphorylation. **(D)** γA3 stub and pseudophosphorylated γA3 stub (γA3 stub SSEE) immunoprecipitates were probed with anti-ubiquitin and anti-GFP. γA3 stub SSEE exhibited a reduced ubiquitin smear as compared to γA3 stub as quantified on the right. **p <0.005.

The 9 amino acid segment contained 2 serines (S759 and S762, numbered according to the full length Pcdh-γA3 sequence from mouse) conserved among the Pcdh-γA family and it remained possible that these serines could be substrates for ubiquitin conjugation (Figure 5A). We therefore mutated these serines to alanine (γA3 stub SSAA) and found that ubiquitination levels and colocalization with ubiquitin (Figure 5B) was similar to the γA3 stub. We thus searched for other activities that could be mediated by this segment and found that when the γA3 stub amino acid sequence was analyzed by NetPhos 3.1, the serines (S759 and S762) were predicted to be potential phosphorylation sites (Figure 5C) with a higher probability than any the other serines in the stub. We therefore created pseudophosphorylated mutants at these sites and studied the effect on ubiquitination and trafficking. We found by quantitative Western blotting that mutation of S759 and S762 to glutamate (γA3 stub SSEE) reduced ubiquitination relative to the γA3 stub (Figure 5D).

### Pseudophosphorylation promotes accumulation at cell junctions

**FIGURE 6:**
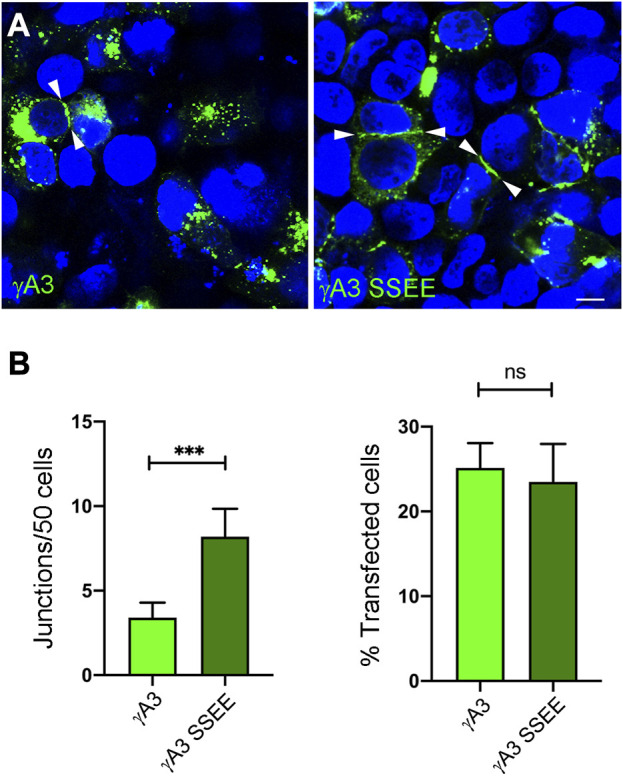
Pseudophosphorylation at S759 and S762 affects trafficking of the full length molecule. **(A)** Cells were transfected with full length Pcdh-γA3 or Pcdh-γA3 SSEE. Junctions between two cells (arrowheads) were identified. **(B)** Quantification of cell junctions per 50 transfected cells. The number of junctions formed by Pcdh-γA3 SSEE was significantly increased relative to wild-type Pcdh-γA3 (left) while the number of transfected cells was equivalent between the 2 constructs (right). Bar = 5 μm ***p <0.001.

Full length Pcdh-γA3 exhibits sparse accumulation at cell-cell junctions in transfected cells which is enhanced by cytoplasmic deletion. We asked whether pseudophosphorylation at S759 and S762 of full length Pcdh-γA3 could promote junction formation (Figure 6A). As compared to wild-type Pcdh-γA3, Pcdh-γA3 SSEE had ∼2 times more junctions per 50 cells even though the efficiency of transfection was similar for both constructs (Figure 6B).

**FIGURE 7:**
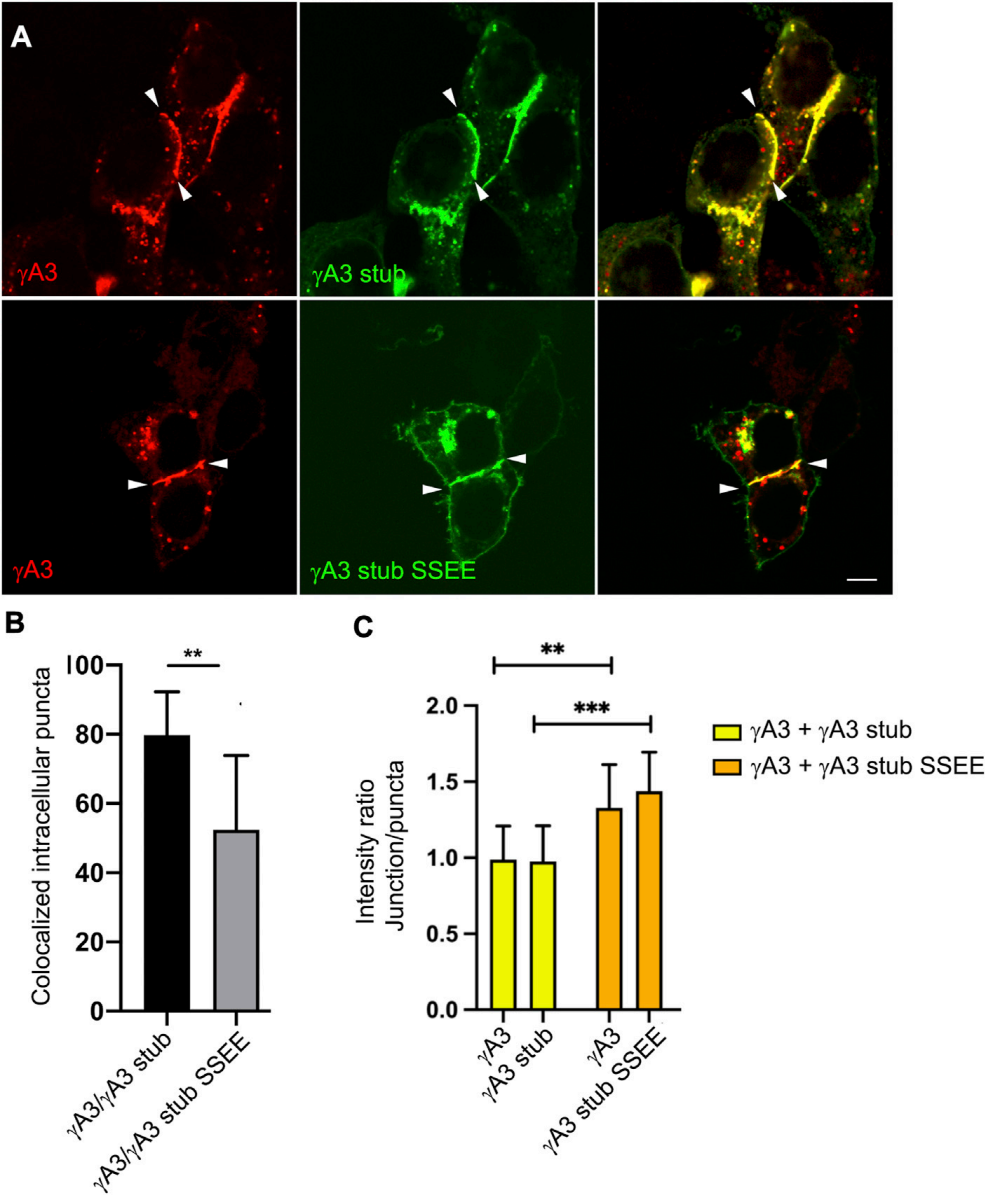
Pseudophosphorylated γA3 stub increases recruitment of wild-type full length Pcdh-γA3 to cell junctions. **(A)** Cells were cotransfected with full length Pcdh-γA3 and either γA3 stub (top) or γA3 stub SSEE (bottom). **(B)** Intracellular puncta colocalization of γA3 stub or γA3 stub SSEE. **(C)** Pcdh-γA3 and cotransfected γA3 stub had a roughly equivalent intensity ratio between junctions and puncta (yellow bars) whereas when Pcdh-γA3 when cotransfected with γA3 stub SSEE both were more intense at the junctions than the intracellular puncta. **p <0.005, ***p <0.001.

Although the γA3 stub lacks the adhesive extracellular domain, it was shown previously that the stub can co-accumulate at cell junctions formed by full length Pcdh-γA3 through interactions involving the VCD (Shonubi et al., 2015). We asked whether the pseudophosphorylation of the stub might alter its colocalization with full length wild type Pcdh-γA3 and influence trafficking of the full-length molecule to cell-cell contacts. When full length Pcdh-γA3 and the γA3 stub were cotransfected, it was found that ∼80% of the intracellular Pcdh-γA3 puncta (not including junctions) colocalized with γA3 stub (Figures 7A, B). In contrast, cotransfection of full length Pcdh-γA3 with γA3 stub SSEE resulted in significantly less colocalization (∼50%) at intracellular puncta (Figures 7A, B). This supports the conclusion that pseudophosphorylation alters intracellular trafficking.

We further asked whether the γA3 stub, when pseudophosphorylated, could alter the distribution of the full length molecule between intracellular puncta and cell-cell contacts. Full length Pcdh-γA3 was nearly equally distributed in signal intensity between junctions and puncta in cell pairs with observable junctions when cotransfected with γA3 stub, yielding a ratio of intensity between junctions and puncta of ∼1 (Figure 7C). This distribution changed when Pcdh-γA3 was cotransfected with γA3 stub SSEE. In this case the full length molecule was more shifted to cell-cell contacts as shown by the higher intensity ratio of junctions to puncta (∼1.4, Figure 7C). Indeed, even the co-trafficking of the stub with the full length molecule shifted to cell-cell contacts when pseudophosphorylated (Figure 7C). Thus, the pseudophosphorylated stub is still able to participate in a cytoplasmic complex with the full length molecule. Overall, the results suggest that a predicted serine phosphorylation site in the VCD influences Pcdh-γA3 ubiquitination and cell surface trafficking.

## Discussion

Pcdhs are emerging as critical players in neurodevelopment and misregulation of the gene cluster or point mutations in the structural genes have been implicated in various neurodevelopmental conditions including autism and schizophrenia reviewed in (Flaherty and Maniatis, 2020). It is therefore critical that we understand how the proteins function in cells. To date their role as adhesion molecules has been firmly established but their exact function seems to vary depending on context and cell type. Their atypical localization in neurons and other cell types point to diverse roles in neurodevelopment. Therefore, cell biological studies could shed important light on their function.

Previous studies have shown that intact Pcdh-γs are inefficiently expressed on the surfaces of many transfected cell types as well as endogenous Pcdh-γs *in vivo*, when compared to classical cadherins (Phillips et al., 2003; Fernández-Monreal et al., 2009; Hanson et al., 2010; O’Leary et al., 2011; Shonubi et al., 2015). Rather it has been found that these proteins are trafficked in the endosomal system, at least in cell lines, but also most likely in neurons both in culture and *in vivo*. The significance of the prominent intracellular trafficking of Pcdh-γs in terms of the function of these proteins is still unknown. This is of particular interest given the different, and perhaps opposing, roles proposed for these proteins in mediating either adhesion at synapses or between dendrites and glial cells, or avoidance of same cell dendrites. It has been proposed that endocytosis and trafficking could regulate the surface activity of Pcdh-γs and alter their adhesion (Buchanan et al., 2010; Phillips et al., 2017). Here we show that the cytoplasmic domain of one Pcdh-γ, Pcdh-γA3, is ubiquitinated and that this activity is influenced by a segment of the cytoplasmic domain that resembles a serine phosphorylation site. Notably, this segment is highly conserved among all 12 Pcdh-γA genes from mouse, except for the Pcdh-γA8 gene, which is a pseudogene.

It is clear from structural studies and cell aggregation experiments that Pcdhs, including Pcdh-γs, have homophilic cell-cell adhesive activity (Obata et al., 1995; Schreiner and Weiner, 2010; Thu et al., 2014; Rubinstein et al., 2015; Rubinstein et al., 2017; Goodman et al., 2016b; Goodman et al., 2017; Brasch et al., 2019). However, early studies on Pcdhs found that the adhesive activity was difficult to detect in the mouse L-cell aggregation assay, when compared to classical cadherins (Obata et al., 1995). Indeed, adhesion was only observed when the Pcdh cytoplasmic domain was replaced with that of E-cadherin, indicating the negative effect on cell adhesion by the Pcdh cytoplasmic domain. Subsequent studies that studied aggregation activity of Pcdhs in various cell lines used cytoplasmic deletions, which were shown to promote the surface localization of Pcdh-γs (Schreiner and Weiner, 2010). Most of the inhibitory effect of the Pcdh cytoplasmic domains on cell adhesion most likely can be attributed to intracellular retention. How and why the Pcdh-γ cytoplasmic domains promote retention in intracellular compartments had not yet been described. We now show for Pcdh-γA3 that ubiquitination of the cytoplasmic domain, in a region encoded by the “variable” γA3 exon (VCD), and not the Pcdh-γ constant domain, is a determinant for intracellular trafficking and is a predicted serine phosphorylation site. Although this region is well-conserved among Pcdh-γA family members, it is different in other Pcdh-γs as well as Pcdh-αs and -βs. Given that the Pcdh-γA family is the largest Pcdh-γ subfamily, and that different Pcdh isoforms can influence surface delivery and adhesive specificity of other isoforms via interaction in cis (Murata et al., 2004; Schreiner and Weiner, 2010; Thu et al., 2014; Rubinstein et al., 2015; Rubinstein et al., 2017; Goodman et al., 2017), it is therefore possible that the segment identified in the present study could be an important regulator of the activity of other members of the Pcdh cluster.

The activity of Pcdh cytoplasmic domains is much less characterized in comparison to other cell adhesion molecules such as the classical cadherins. Most attention has been focused on the constant cytoplasmic domains present in the Pcdh-α and Pcdh-γ clusters. The earliest yeast two hybrid studies found that Pcdh-α proteins could interact with Fyn (Kohmura et al., 1998) although it is not known if members of the β and γ clusters also interact with Fyn. Other yeast two hybrid studies revealed that the constant cytoplasmic domains of both the γ and α clusters interact with the tyrosine kinases Pyk2 and FAK (Chen et al., 2009) as well as PDCD10 (Lin et al., 2010). A screen for altered signaling pathways in Pcdh-γ disrupted forebrain showed that a signaling pathway involving FAK, PKC and MARCKS kinases was overactive in Pcdh-γ mutants and that this resulted in defective dendrite morphogenesis (Garrett et al., 2012). Rac1 was shown to be involved in this pathway as its overexpression could rescue defective dendritic morphogenesis (Suo et al., 2012). PKC was later found to directly phosphorylate the Pcdh-γ constant cytoplasmic domain (Keeler et al., 2015) and this was a requirement for proper dendrite arborization. It was also shown that deletion of the constant domain only could promote increased cell-cell junctions (Fernández-Monreal et al., 2009) but that trafficking to endosomes required deletion of the constant and VCD domains (O’Leary et al., 2011). It is possible that ubiquitination of the VCD of the Pcdh-γA family could influence signals mediated by the constant domain by altering the localization of Pcdh-γAs, which could, in turn alter trafficking of other Pcdhs through cis interactions.

Less information is known about how the VCDs influence Pcdh signaling function, even though these comprise almost one-half of the cytoplasmic domain. Recently, a role for the VCD of Pcdh-γC3, an isoform that is more widely expressed in neurons, in interaction with Axin 1, resulted in inhibition of the canonical Wnt pathway (Mah et al., 2016). This interaction was found to be necessary to promote dendritic complexity in cultured neurons (Steffen et al., 2023). The Pcdh-γC3 VCD is unique in that it does not resemble that of other Pcdhs. In contrast, in the Pcdh-γA family, the VCD shows significant conservation.

These results in cell culture agree well with the observed distribution of endogenous Pcdhs in apparently similar compartments in cultured neurons and tissue *in vivo* (Phillips et al., 2003; Fernández-Monreal et al., 2010; LaMassa et al., 2021). In particular, Pcdh-γs were found in dendrites and spine compartments but their role in these locations in terms of synaptic function remain unknown. Pcdh-γ localization was an indicator of synaptic maturation (LaMassa et al., 2021), however. Interaction of neurons and astrocytes via Pcdh-γs was shown to promote synaptogenesis (Garrett and Weiner, 2009), but their adhesive role at the synaptic cleft remains unknown in terms of whether the molecules promote or inhibit synaptic contacts. Recent studies using FRET to detect cell-cell interaction of Pcdh-γB2 have confirmed its localization in cell bodies, dendrites and spine compartments where they appear to be intracellular and not engaged in homophilic interaction at synapses (Hoshino et al., 2023). Others have found that Pcdh-γs can have an inhibitory effect on the number of dendritic spines by negatively regulating neuroligin-1 (Molumby et al., 2017; Steffen et al., 2021), although it has not been determined if this involves cell-cell adhesive activity of the Pcdh-γs. In contrast, Pcdh-γs have a positive effect on the formation of synapses between sensory axons and spinal cord neurons (Meltzer et al., 2023), attributable to adhesive interactions of the Pcdh-γC3 isoform. It may be that the differences on synaptic development observed in various systems is due to different activities of Pcdh-γ isoforms and further studies will be needed to characterize the role of these isoforms on synaptic development.

Receptor ubiquitination is a known factor to induce endocytosis and intracellular trafficking of signaling receptors in neurons and at the synapse [Reviewed in (Folci et al., 2020)]. In addition, other cell adhesion molecules can be ubiquitinated and this has been shown to be an important regulator of cell adhesion. Pcdh-αs and Pcdh-γs were both shown to undergo endocytosis and trafficking in the endosomal system in neuronal cell lines (Buchanan et al., 2010) that could be a consequence of ubiquitination. Our results show that Pcdhs have a propensity for ubiquitination and trafficking in endosomes, which could be associated with lysosomal degradation and, hence, downregulation of these molecules. This may indicate that these molecules are particularly dynamic mediators of cell-cell interaction that need to be precisely tuned at various points in neural development and such regulation may account for the diverse roles observed for these molecules.

## Materials and methods

### Cell culturing and transfection

HEK 293 cells were cultured as described (Fernández-Monreal et al., 2009). Seeded HEK 293 cells were grown for 24 h on 6 well plates for immunoprecipitation or on glass coverslips for microscopy and then transfected using Lipofectamine 2000, following the manufacturer’s protocol. Cells were processed the next day for immunoprecipitation or immunostaining as below.

### DNA constructs

Full length mouse Pcdh-γA3-GFP, -RFP, cytoplasmic deletions and γA3 stub-GFP constructs have been described previously (Fernández-Monreal et al., 2010; Hanson et al., 2010; O’Leary et al., 2011; Shonubi et al., 2015). γA3 stub-RFP and new γA3 stub deletions from the C-terminus in the γA3 stub backbone were generated by PCR and subcloning. Point mutations within the γA3 stub and full length Pcdh-γA3 were generated using the Agilent QuikChange Site Directed Mutagenesis Kit (Agilent Technologies). GFP-Rab5B was a gift from Gia Voeltz (Addgene plasmid # 61802; http://n2t.net/addgene:61802; RRID:Addgene_61802), GFP-Rab7A was a gift from Gia Voeltz (Addgene plasmid # 61803; http://n2t.net/addgene:61803; RRID:Addgene_61803), GFP-rab11 WT was a gift from Richard Pagano (Addgene plasmid # 12674; http://n2t.net/addgene:12674; RRID:Addgene_12674), mRFP-Ub was a gift from Nico Dantuma (Addgene plasmid # 11935; http://n2t.net/addgene:11935; RRID:Addgene_11935).

### Immunoprecipitation and Western blotting

24 h post-transfection, cells were washed with ice cold PBS and lysed with 1.2 mL ice cold RIPA buffer 1% Triton, 0.5% Sodium Deoxycholate, 150 mM NaCl, 0.1% SDS, 50 mM Tris pH7.7, 2 mM EDTA, 10 mM N-Ethylmaleimide, and protease inhibitor (Complete Mini, Roche) on ice for 20 min with agitation. Lysate was centrifuged and supernatant was incubated with 20 μL of anti-GFP agarose (MBL International Cat# D153-8, RRID:AB_591815). The beads were washed and eluted with SDS-PAGE sample buffer.

Samples were loaded on 10% SDS-PAGE gels, transferred to nylon membranes, blocked and probed with polyclonal antibodies to GFP (Abcam Cat# ab290, RRID:AB_303395) and mouse anti-ubiquitin (Thermo Fisher Scientific Cat# 13-1600, RRID:AB_2533002). Secondary detection employed IRDye 800CW goat anti-rabbit LI-COR Biosciences Cat# 926-32211, RRID:AB_621843) and IRDye 680RD goat anti-mouse (LI-COR Biosciences Cat# 926-68072, RRID:AB_10953628) using the LI-COR Odyssey system. For the Western blot quantification, a minimum of three experiments were run and analyzed using ImageJ. The amount of ubiquitin smear and its intensity was quantified in relation to the total amount of the GFP band.

### Confocal microscopy and quantification

Cells on coverslips were fixed in 4% paraformaldehyde, 4% sucrose in PBS, washed in PBS and mounted. Single plane images were acquired on a Leica SP2 confocal microscope (Advanced Imaging Facility, College of Staten Island).

ImageJ was used to quantify images. At least 10 images at 63X from an individual experiment were used to quantify the degree of colocalization between Pcdh-γA3-GFP and ubiquitin-RFP constructs. Red and green channels were thresholded and the total area of the intracellular GFP puncta was measured using the Nucleolus Counter plugin. Thresholded red and green channels were merged and colocalized area was measured in pixels. The area in pixels of colocalized puncta was divided by the total area of γA3 GFP and multiplied by 100 to find the degree of colocalization. Results were analyzed by *t*-test. The same approach was used to analyze trafficking of Pcdh-γA3-RFP constructs and mutants to the Rab5, Rab7, and Rab11 positive endosomes.

To quantify the number of junctions formed by Pcdh-γA3 and Pcdh-γA3 SSEE, an equal number of cells were seeded to form a dense monolayer and cells were transfected. Images were captured at ×40 magnification and analyzed using ImageJ. Images were thresholded and nuclei were counted to determine the total cell number per image. All transfected cells were counted and the transfection efficiency was calculated as a percentage. The number of junctions were counted for each transfected cell from at least 10 images and data was normalized to find the number of junctions per 50 transfected cells.

Quantification of the relative intensity of puncta to junctions was as follows. Images of paired cells were captured at ×63 magnification. Each punctum in a cell has a distinct pixel intensity. The brightness of a puncta indicates the level of fluorescence protein present. To determine the total pixel intensity of only colocalized puncta and junctions, the red and green channels were merged, and thresholded. ImageJ’s image calculator was utilized to extract the colocalized area exclusively. Then only colocalized areas containing junctions or puncta were selected, and their coordinates were transferred to the ROI in ImageJ. The saved area coordinates then were transferred to the original red and green channel images to determine the pixel intensity level for each area individually. Total average pixel intensity was calculated separately once the dataset representing the area of interest was normalized based on its size. All areas of interest were divided by 100 and then multiplied by their corresponding intensity. This process enabled the calculation of the intensity ratio between junctions and puncta for each channel separately, representing the relative brightness of puncta versus junctions. By following this protocol, the mean pixel intensity of the colocalized area was determined for multiple channels separately. At least 10-12 images with 2-8 cells per image were quantified to get the average for each condition. Results were analyzed by *t*-test.

## Data Availability

The raw data supporting the conclusion of this article will be made available by the authors, without undue reservation.
